# NDRG1 promotes growth of hepatocellular carcinoma cells by directly interacting with GSK-3β and Nur77 to prevent β-catenin degradation

**DOI:** 10.18632/oncotarget.4913

**Published:** 2015-08-20

**Authors:** Wen-Jing Lu, Mei-Sze Chua, Wei Wei, Samuel K. So

**Affiliations:** ^1^ Asian Liver Center, Department of Surgery, Stanford University School of Medicine, Stanford, CA 94305, USA

**Keywords:** NDRG1, β-catenin, GSK-3β, Nur77, liver cancer

## Abstract

The N-myc downstream regulated gene 1 (NDRG1) is significantly associated with advanced tumor stages and poor survival of hepatocellular carcinoma (HCC), thereby implicating it as a potential target for HCC treatment. We aim to further understand its biological roles in hepatocarcinogenesis, as a means to exploit it for therapeutic purposes. By screening using the ProtoArray^®^ Human Protein Microarrays, we identified glycogen synthase kinase 3β (GSK-3β) and the orphan nuclear receptor (Nur77) as potential interaction partners of NDRG1. These interactions were confirmed in HCC cell lines *in vitro* by co-immunoprecipitation; and co-localizations of NDRG1 with GSK-3β and Nur77 were observed by immunofluorescence staining. Additionally, high levels of NDRG1 competitively bind to GSK-3β and Nur77 to allow β-catenin to escape degradation, with consequent elevated levels of downstream oncogenic genes. *In vivo,* we consistently observed that NDRG1 suppression in HCC xenografts decreased β-catenin levels and its downstream target Cyclin D1, with concomitant tumor growth inhibition. Clinically, the over-expression of NDRG1 in HCC patient samples is positively correlated with GSK-3β-9ser (│R│= 0.28, *p* = 0.01), Nur77 (│R│= 0.42, *p* < 0.001), and β-catenin (│R│= 0.32, *p* = 0.003) expressions. In conclusion, we identified GSK-3β and Nur77 as novel interaction partners of NDRG1. These protein-protein interactions regulate the turnover of β-catenin and subsequent downstream signaling mediated by β-catenin in HCC cells, and provides potential targets for future therapeutic interventions.

## INTRODUCTION

Hepatocellular carcinoma (HCC) is the seventh most lethal malignancy worldwide, and the second leading cause of cancer related death (746,000 in 2012) (2012 Globocan (WHO IARC)). It is typically fatal with a mortality-to-incidence ratio of 0.95 due to difficulties in early detection, limited therapeutic options, and high recurrence rates. Lack of reliable HCC biomarkers leads to late diagnosis, especially in high risk populations (chronic carriers of hepatitis B virus or hepatitis C virus). Meanwhile, there is no curative therapy for the majority of HCC patients who are diagnosed at advanced stages.

Our previous gene expression data identified N-Myc downstream regulated gene 1 (NDRG1) as a clinically relevant biomarker of HCC. NDRG1 is a stress response protein whose expression is regulated by a variety of cellular stresses, especially hypoxia [1–4]. Significant up-regulation of NDRG1 was observed in HCC tissues compared to adjacent non-tumor liver tissues at both mRNA and protein levels [5, 6], and it has also been suggested as a novel biomarker of liver cancer recurrence [7]. More importantly, over-expression of NDRG1 was significantly and positively associated with aggressive tumor features such as high AFP levels (400 ng/ml), advanced stages, vascular invasion, dedifferentiation, metastasis, recurrence, and poor survival rates [6]. Recent functional studies suggested that knockdown of NDRG1 induced cellular senescence through inhibiting GSK-3β–p53/p16 pathway in HCC cells, implying a possible connection of NDRG1 with the Wnt/β-catenin signaling pathway which plays crucial roles in many types of malignancies, including HCC [8]. These clinical and functional observations suggest that NDRG1 is a potential therapeutic target for HCC.

We aim to further elucidate the biological functions mediated by NDRG1 in HCC cells, so as to improve our understanding of hepatocarcinogenesis and tumor progression, which in turn may allow rational design of novel therapeutic approaches targeting NDRG1. To this end, we attempt to identify novel interaction partners of NDRG1 through protein array screening and to further study the molecular events regulated by these interactions in HCC cells.

## RESULTS

### NDRG1 binds directly to GSK-3β and Nur77 in HCC cells

**Figure 1:**
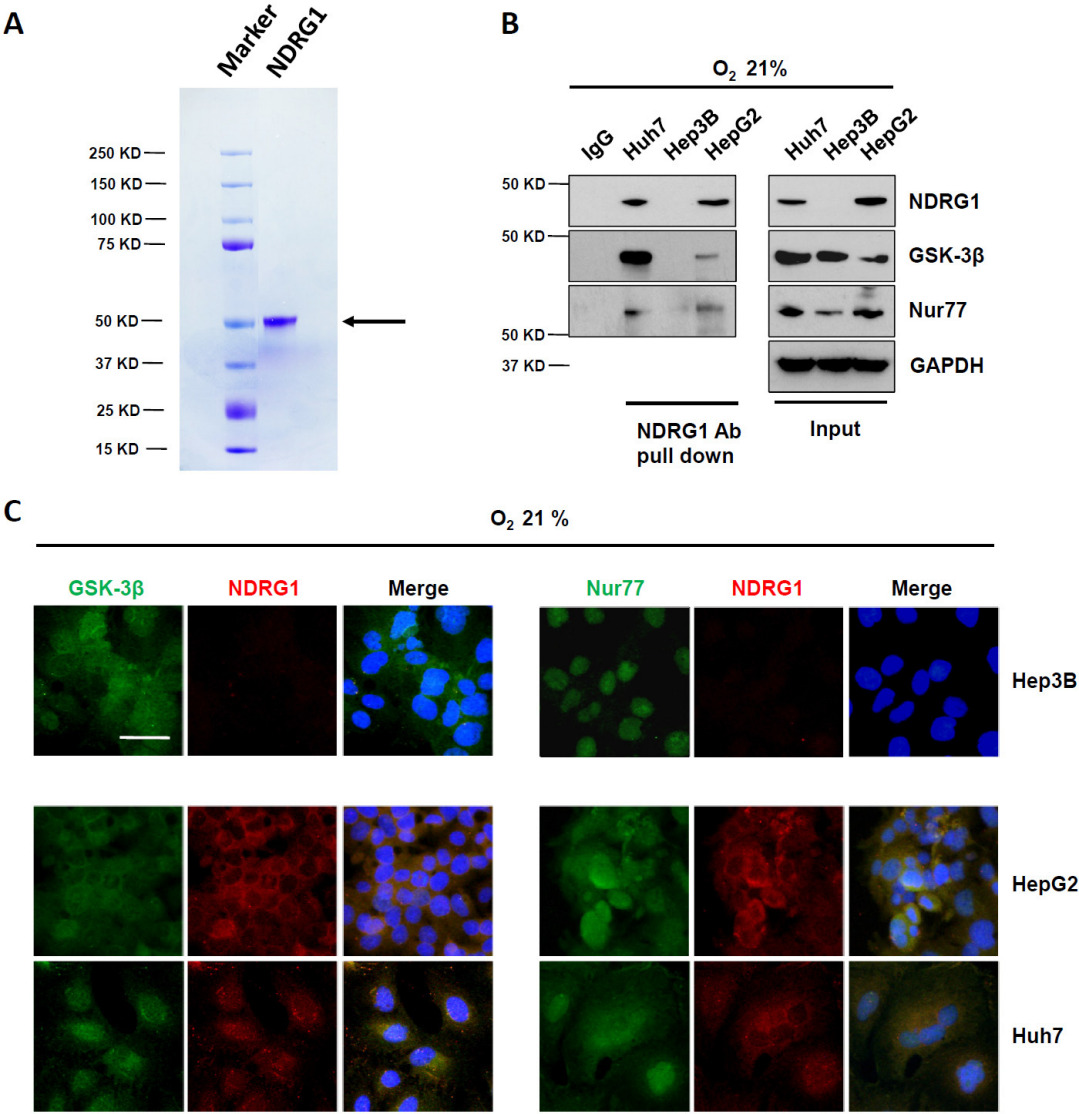
NDRG1 binds to GSK-3β and Nur77 in HepG2 and Huh7 cells **A.** SDS-PAGE gel analysis of purified NDRG1 (indicated by arrow). **B.** Pull-down assay demonstrating the interaction between NDRG1 and GSK-3β, and between NDRG1 and Nur77 in Huh7 and HepG2 cells with endogenous levels of NDRG1, but not in Hep3B cells, which lacks NDRG1 expression under normoxia. **C.** Immunofluorescence staining showing the co-localization of NDRG1 with GSK-3β or with Nur77 in Huh7 and HepG2 cells, but not in Hep3B cells under normoxia. DAPI was used to stain the nucleus (400× magnification; scale bars: 5 μm).

To better understand the role of NDRG1 in hepatocarcinogenesis, we first purified NDRG1 full length protein with the V5 tag. Coomassie gel staining showed a single band at 50 KD, representative of NDRG1 (arrow; Figure 1A). When this purified full length NDRG1 protein was used to probe for potential interaction proteins using the protein microarray, we identified a list of candidate proteins with *p* values less than 0.05. Among these candidates, we selected GSK-3β and Nur77, two functionally important proteins on the top of list, for further investigations. To validate these potential hits, we first confirmed the interactions of NDRG1 with GSK-3β and Nur77 in a panel of HCC cell lines using Co-IP. The interactions of NDRG1 with GSK-3β, and of NDRG1 with Nur77 were only detected in Huh7 and HepG2 cells, but not in Hep3B cells which lack NDRG1 expression under normoxia (Figure 1B). Furthermore, immunofluorescence staining consistently showed co-localization (yellow signal) of NDRG1 with GSK-3β, and of NDRG1 with Nur77 in Huh7 and HepG2 cells, but not in Hep3B cells (Figure 1C).

**Figure 2:**
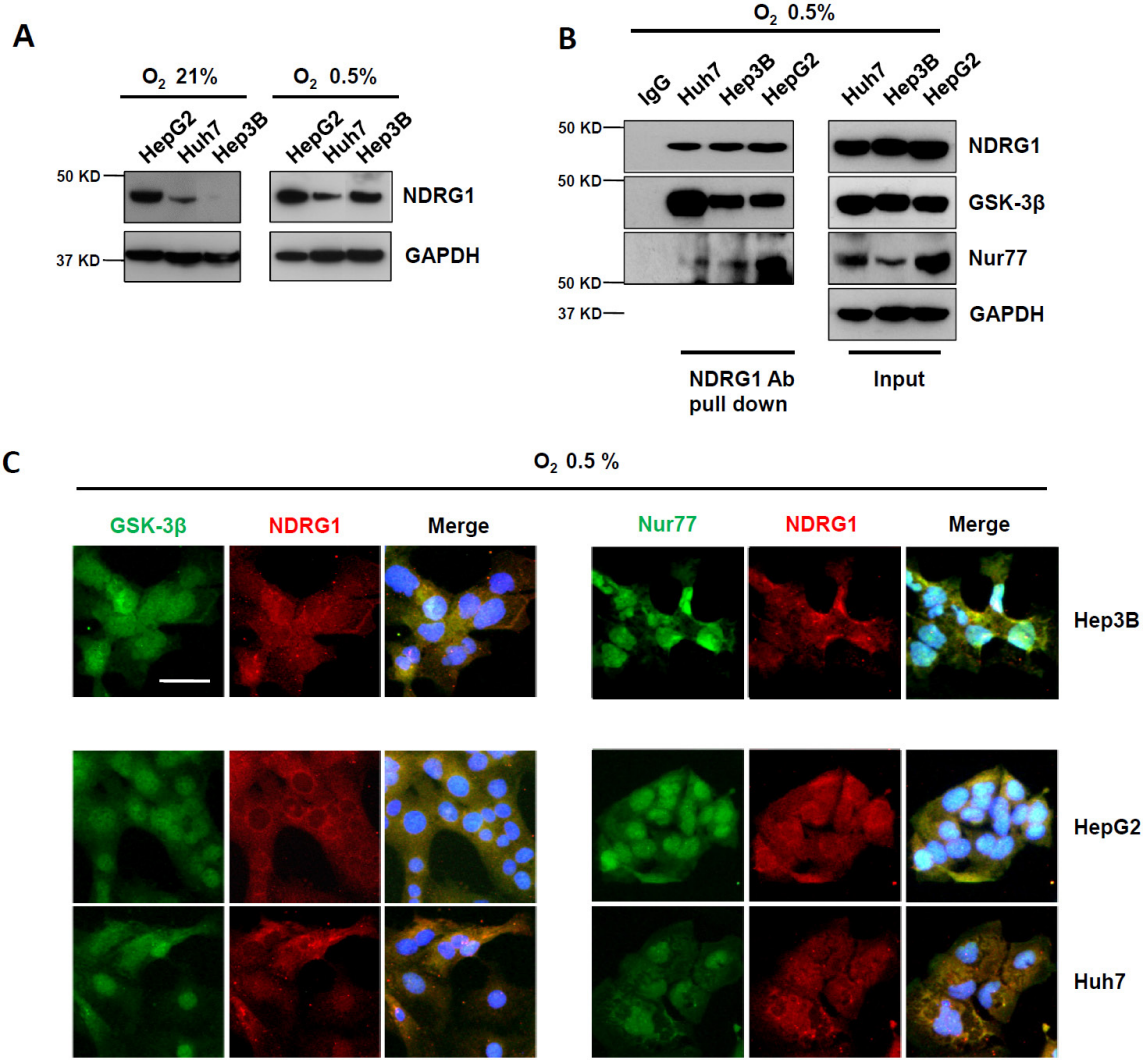
Hypoxia-inducible NDRG1 expression and its interactions with GSK-3β and Nur77 in HCC cells **A.** Western blot showing that NDRG1 expression was increased under hypoxia (0.5% O_2_) in HCC cells. GAPDH was used as loading control. **B.** Co-IP showing interaction of NDRG1 with GSK-3β and with Nur77 in HCC cells under hypoxia. **C.** Immunofluorescence staining showed the co-localization of NDRG1 and GSK-3β or Nur77 in HCC cells under hypoxia (400× magnification; scale bars: 5 μm).

When HCC cells were cultured under hypoxic conditions of 0.5% O_2_, NDRG1, a known hypoxia-inducible protein, was induced in all three cell lines, with marked induction observed in Hep3B cells that had undetectable levels of NDRG1 under normoxia (Figure 2A). Using Co-IP, we again detected interaction of NDRG1 with GSK-3β, and of NDRG1 with Nur77 in all three cell lines under hypoxia (Figure 2B). Sub-cellular co-localizations of NDRG1 with GSK-3β or with Nur77 were again consistently observed by immunofluorescence staining under hypoxia in all three cell lines (Figure 2C). These observations validate that NDRG1 binds directly with GSK-3β and Nur77 in HCC cells, and these interactions are maintained/induced under hypoxic conditions, which may mediate the functions of NDRG1 in HCC cells particularly under the hypoxic tumor microenvironment.

### NDRG1 regulates nuclear accumulation of β-catenin in HCC cells

Both GSK-3β and Nur77 mediate β-catenin degradation through independent pathways [9, 10]. Thus, we hypothesized that the interaction of NDRG1 with either or both of these interaction partners may be associated with β-catenin regulation in HCC cells. We used HepG2 and Hep3B cells as our working models, in particular inducing NDRG1 in Hep3B cells under hypoxia to support data observed in HepG2 cells (under normoxia and hypoxia).

**Figure 3:**
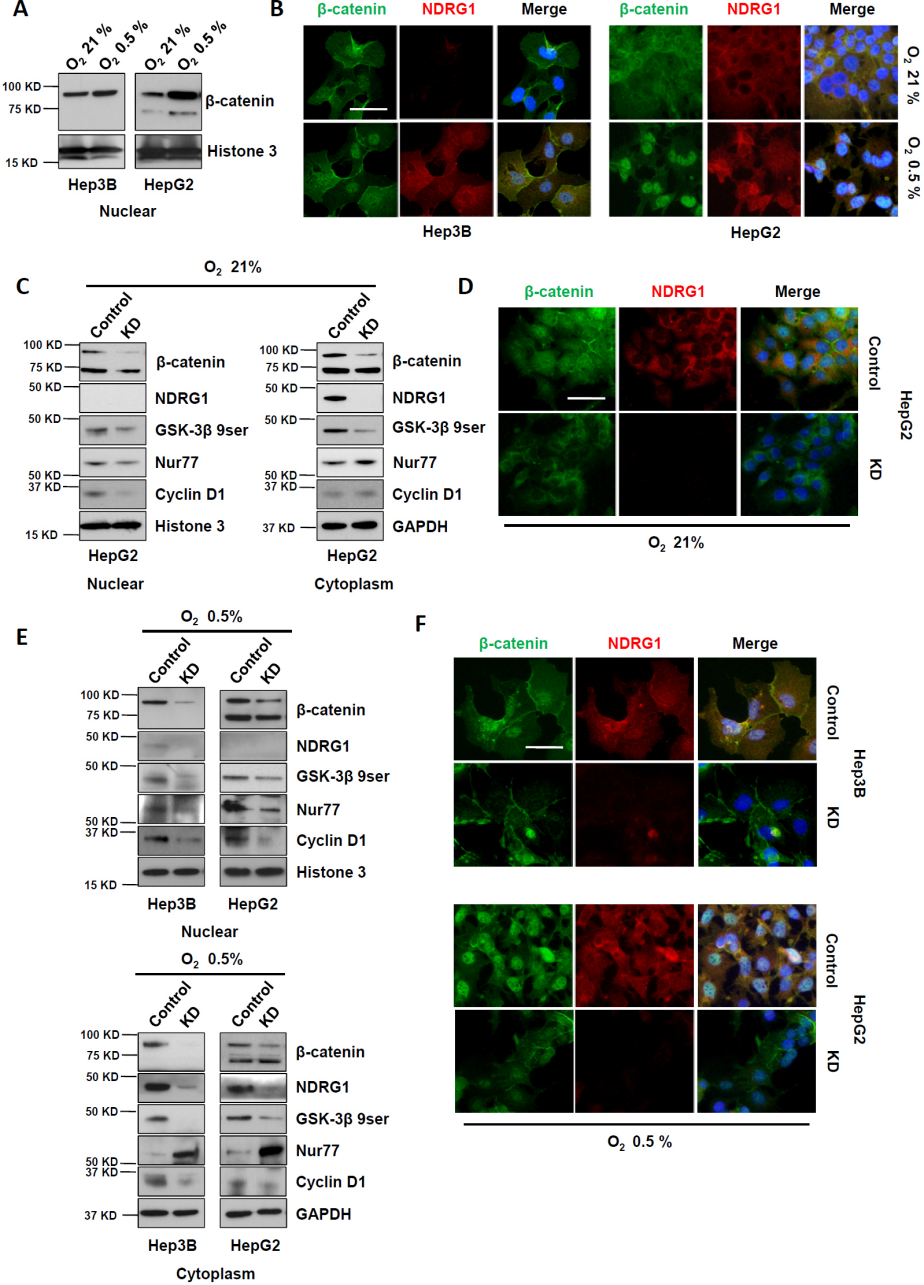
Suppression of NDRG1 by siRNA prevented β-catenin nuclear accumulation in HCC cells **A.** Western blot and **B.** immunofluorescence showing expression and localization of NDRG1 and β-catenin in Hep3B and HepG2 cells, under normoxia and hypoxia (400× magnification; scale bars: 5 μm). **C.** Western blot showing that suppression of NDRG1 decreased cytoplasmic and nuclear β-catenin levels, with corresponding changes in GSK-3β 9ser, Nur77, and Cyclin D1. GAPDH and Histone3 are used as loading controls. **D.** The changes in NDRG1 and β-catenin protein levels and their cellular localizations (after NDRG1 suppression) were confirmed by immunofluorescence staining (400× magnification; scale bars: 5 μm). **E.** The changes in protein levels of NDRG1, β-catenin, GSK-3β 9ser, Nur77, and Cyclin D1 caused by suppression of NDRG1 under hypoxia were detected by Western blot. GAPDH and Histone3 are used as loading controls. **F.** The changes in NDRG1 and β-catenin protein levels and their cellular localizations (after NDRG1 suppression) were confirmed by immunofluorescence staining of HCC cells cultured under hypoxia (400× magnification; scale bars: 5 μm).

Using Western blot and immunofluorescence staining following subcellular fractionation, we observed that nuclear localization of β-catenin was enhanced in both HepG2 and Hep3B cells under hypoxia, corresponding to induction of NDRG1 in both cell lines (Figure 3A and 3B). These data indicated that hypoxia promoted nuclear accumulation of β-catenin in HCC cells, which may be mediated by hypoxia-inducible NDRG1 expression. Thus, high levels of NDRG1 may promote nuclear accumulation of β-catenin in HCC cells.

To demonstrate whether NDRG1 is involved in the regulation of β-catenin nuclear accumulation in HCC cells, the NDRG1-specific siRNA was transfected into HepG2 cells which express high levels of NDRG1 even under normoxia. Western blot results indicated that suppression of NDRG1 expression reduced nuclear accumulation of β-catenin, accompanied by reduced levels of GSK-3β 9ser (inactive form of GSK-3β), and Cyclin D1 (a key downstream target of β-catenin) (Figure 3C). NDRG1 suppression also enhanced cytoplasmic Nur77 levels, which might also act to further degrade β-catenin (Figure 3C). Immunofluorescence staining consistently showed that NDRG1 suppression dramatically decreased β-catenin levels and prevented its nuclear accumulation (Figure 3D).

To determine whether the hypoxia-induced nuclear accumulation of β-catenin is regulated by NDRG1, we transfected HepG2 and Hep3B cells with NDRG1-specific siRNA and cultured these cells under hypoxia. Subcellular fractionation followed by Western blotting showed that NDRG1 suppression decreased nuclear accumulation of β-catenin in both HepG2 and Hep3B cells under hypoxia, with accompanying decreases in GSK-3β 9ser and Cyclin D1 levels, and increased cytoplasmic accumulation of Nur77 (Figure 3E). Consistently, immunofluorescence results demonstrated markedly reduced cytoplasmic and nuclear β-catenin levels after NDRG1 suppression (Figure 3F).

### Interaction of NDRG1 with GSK-3β or Nur77 regulates β-catenin degradation in HCC cells

**Figure 4:**
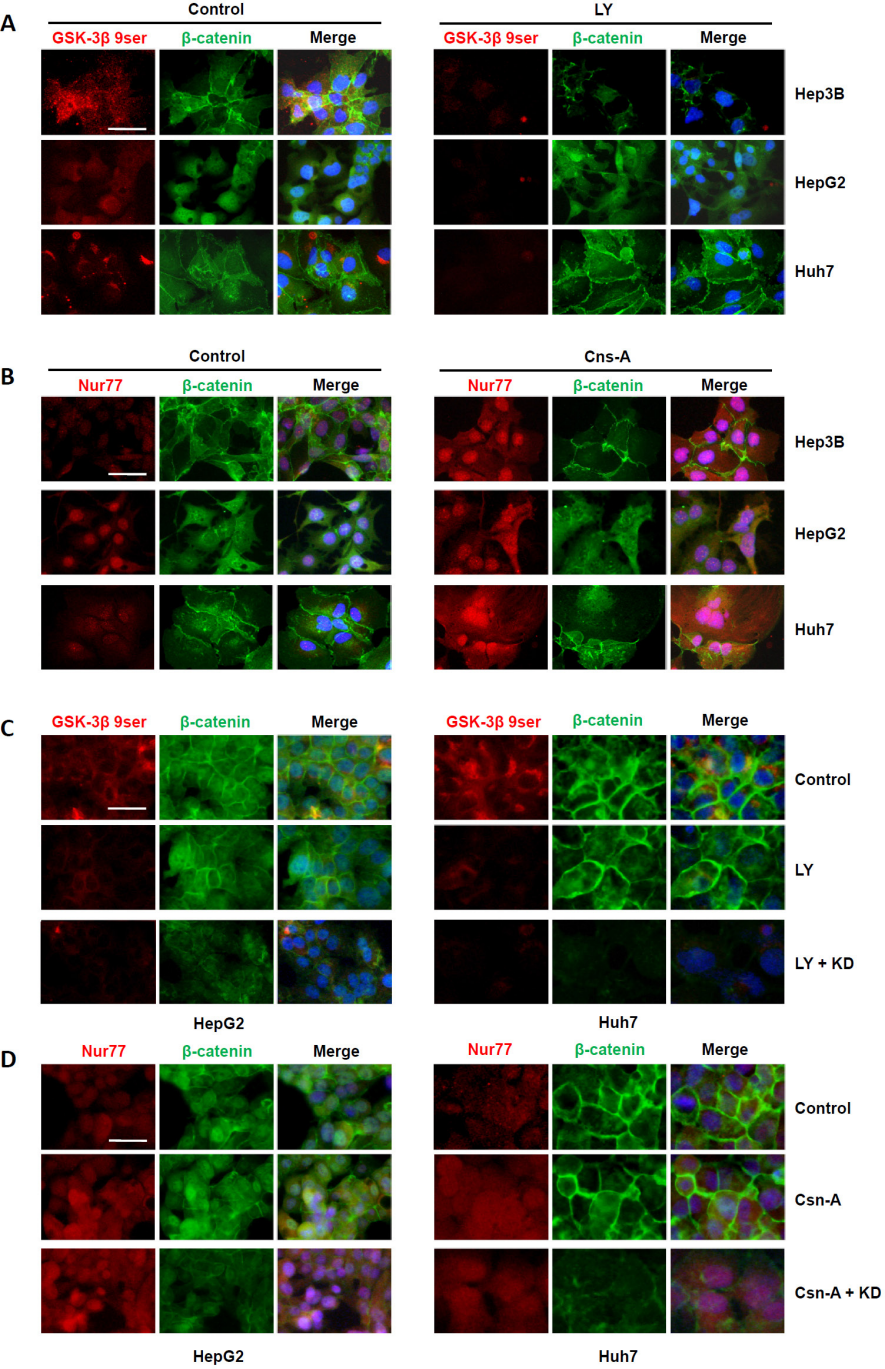
Interaction of NDRG1 with GSK-3β or Nur77 regulates β-catenin degradation in HCC cells **A.** Treatment with either the GSK-3β agonist (LY294002; LY) or **B.** with the Nur77 agonist (Cns-A) decreased β-catenin levels in Hep3B cells only, but not in HepG2 and Huh7 cells, under normoxia where Hep3B cells lack NDRG1 expression. (**C.** and **D.**) When HepG2 cells and Huh7 cells were pre-treated with NDRG1 siRNA, both agonists caused decreased levels of β-catenin respectively (400× magnification; scale bars: 5 μm).

Since both GSK-3β and Nur77 facilitate β-catenin degradation, and we observed that NDRG1 enhance β-catenin accumulation, we further hypothesized that NDRG1 interaction with GSK-3β and Nur77 may impede β-catenin degradation (allowing nuclear accumulation) in HCC cells. To test this hypothesis, we treated Hep3B cells (under normoxia; no NDRG1) with 10 μM of the GSK-3β agonist LY294002, or the Nur77 agonist Cns-A for 48 hours, and then analyzed β-catenin expression and localization in HCC cells. LY294002 enhanced GSK-3β activity as observed by decreased levels of GSK-3β 9ser (the inactive form of GSK-3β), whereas Cns-A up-regulated Nur77 expression and also its nuclear expression in all three HCC cell lines. Even though both these events would be expected to promote β-catenin degradation, we observed reduced β-catenin only in Hep3B cells which have undetectable NDRG1 expression under normoxia (Figure 4A and 4B). These data suggested that the presence of high NDRG1 levels in the other two cell lines (HepG2 and Huh7) may have a protective effect against β-catenin degradation.

To further confirm this observation, we transfected HepG2 and Huh7 cells (with high endogenous NDRG1 levels) with NDRG1-specific siRNA, and examined the effects of NDRG1 protein suppression on GSK-3β or Nur77 agonist treatment under normoxia. As expected, NDRG1 suppression reduced β-catenin levels and its nuclear accumulation (Figure 4C and 4D), similar to what was observed in Hep3B cells. This was consistently observed in Hep3B cells (Supplementary Figure 1A and 1B). Under normoxia, treatment with either GSK-3β or Nur77 agonist alone decreased β-catenin level markedly. However, under hypoxia when NDRG1 was induced, treatment with either agonist alone caused only a minimal decrease in β-catenin level. Marked decrease in β-catenin level was observed only when hypoxic Hep3B cells were treated with either agonist combined with NDRG1 siRNA. These data demonstrate the protective role of NDRG1 against in β-catenin degradation.

### NDRG1 competitively disrupts GSK-3β-β-catenin and Nur77-β-catenin interactions in HCC cells

**Figure 5:**
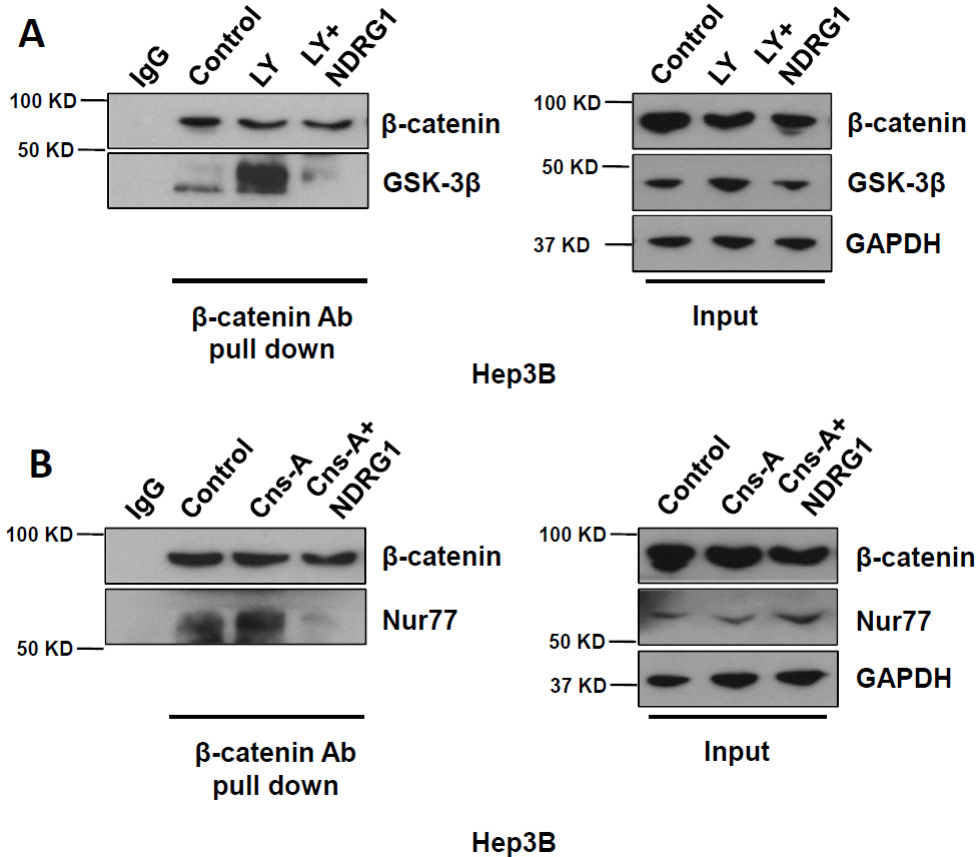
NDRG1 competitively disrupts GSK-3β - β-catenin and Nur77 - β-catenin interactions in HCC cells **A.** and **B.** Co-IP was used to assess GSK-3β - β-catenin and Nur77 - β-catenin interactions in Hep3B cells under normoxia (absence of NDRG1), when treated with either GSK-3β agonist (LY) alone, with Nur77 agonist (Cns-A) alone, or after the addition of purified NDRG1 protein. Loading ratio of input/Co-IP = 1:10.

To further understand the protective effect of NDRG1 against β-catenin degradation through its interactions with GSK-3β and Nur77, we examined the possibility that NDRG1 might disrupt GSK-3β-β-catenin and Nur77-β-catenin interactions through competitive binding. We treated Hep3B cells with either GSK-3β agonist LY294002, or Nur77 agonist Cns-A, and then used β-catenin antibody to pull down the complexes, which were detected using either GSK-3β or Nur77 respectively. We observed that each agonist alone enhanced the binding of GSK-3β to β-catenin (Figure 5A) and of Nur77 to β-catenin (Figure 5B). The addition of purified NDRG1 protein (2 μg) competitively blocked these interactions (Figure 5A and 5B). These data suggest that increased NDRG1 expression can enhance β-catenin functions in HCC cells by preventing its degradation through competitive inhibitory binding with GSK-3β and Nur77.

### Suppression of NDRG1 *in vivo* decreased β-catenin nuclear accumulation and decreased xenograft growth

**Figure 6:**
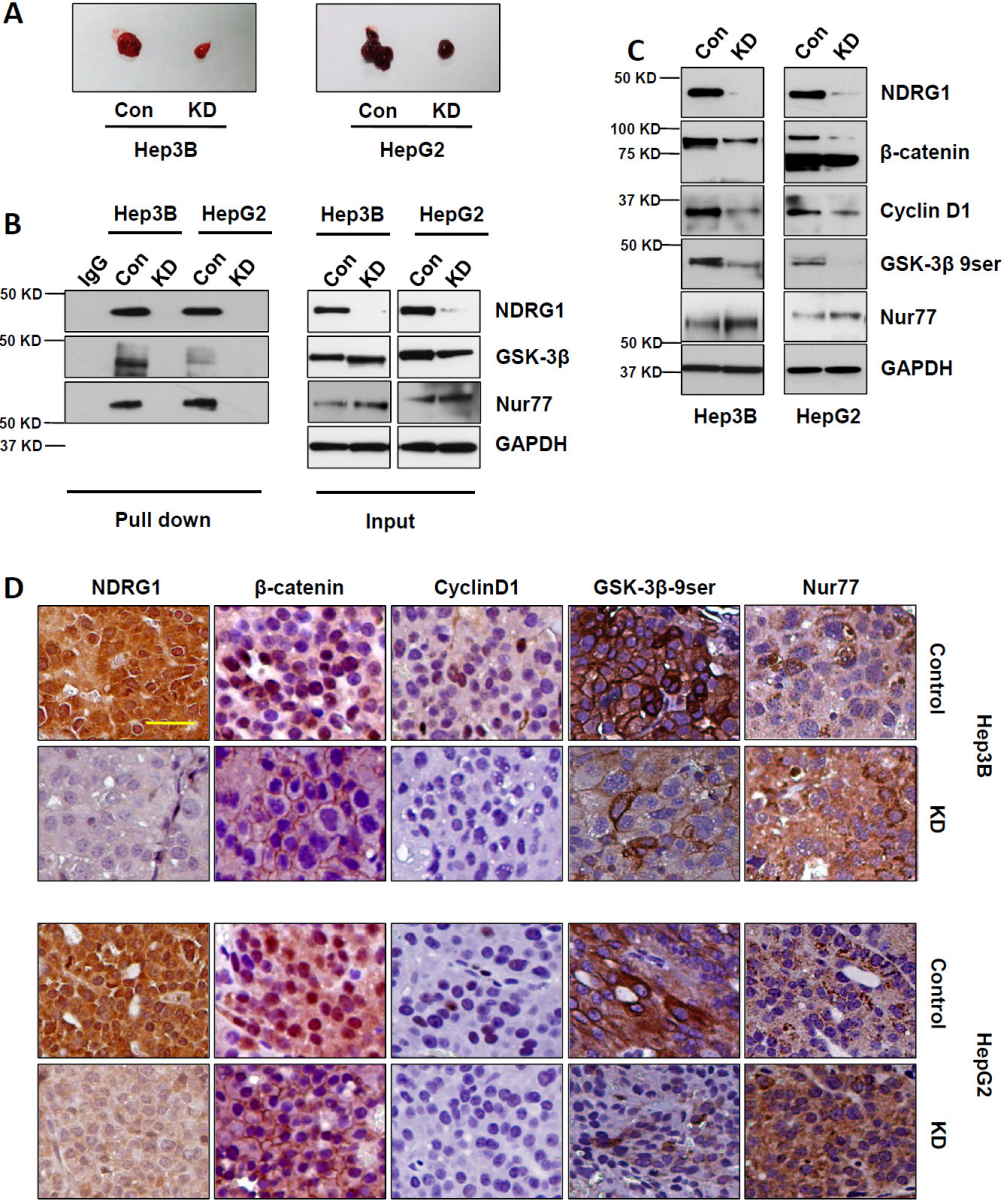
Suppression of NDRG1 *in vivo* decreased β-catenin nuclear accumulation and decreased xenograft growth **A.** Comparison of harvested tumor size from mice bearing HepG2 and Hep3B xenografts expressing the control (con) or NDRG1 shRNA (KD) vectors. Representative images in each group showing that NDRG1 suppression led to smaller tumor sizes four weeks after cell inoculation. **B.** GSK-3β-β-catenin and Nur77-β-catenin protein interactions were detected in tumor lysates in control groups in both Hep3B and HepG2 xenografts, but not in the NDRG1 shRNA groups. **C.** Representative Western blot images showing the protein levels of NDRG1, β-catenin, CyclinD1, GSK-3β 9ser, and Nur77. **D.** Immunohistochemical staining showing the localization and expression of NDRG1, β-catenin, and Cyclin D1, GSK-3β 9ser, and Nur77 in xenograft tumor sections (200× magnification; scale bars: 10 μm).

To further confirm the role of NDRG1-GSK-3β and NDRG1-Nur77 interactions *in vivo*, we inoculated Hep3B and HepG2 cells carrying inducible control or NDRG1 shRNA expression cassettes subcutaneously into nude mice. After 4 weeks, xenografts derived from NDRG1 shRNA cells were significantly smaller than those from control shRNA cells (Figure 6A). Using Co-IP, we detected NDRG1-GSK-3β and NDRG1-Nur77 interaction complexes in tumor lysates of control groups from both Hep3B and HepG2 xenografts, but not in their respective NDRG1 shRNA groups (Figure 6B). Since the solid tumors are expected to be hypoxic, Hep3B xenografts represent Hep3B cells cultured under hypoxia, and therefore also have high levels of NDRG1 with corresponding interactions with GSK-3β and Nur77.

Using Western blot, we observed expected decreased levels of β-catenin and its downstream target gene Cyclin D1 after NDRG1 knockdown in the xenografts (Figure 6C). Correspondingly, the levels of GSK-3β-9ser were decreased (indicating enhanced GSK-3β activity), while Nur77 levels were increased, which might account for enhanced β-catenin degradation when NDRG1 was suppressed. Immunohistochemistry verified these results, showing that NDRG1 suppression decreased β-catenin expression and prevented its nuclear accumulation, with concomitant reductions of Cyclin D1, GSK-3β 9ser, and enhanced Nur77 (Figure 6D).

### Correlation of NDRG1, GSK-3β, Nur77, and β-catenin levels in HCC patient specimens

**Figure 7:**
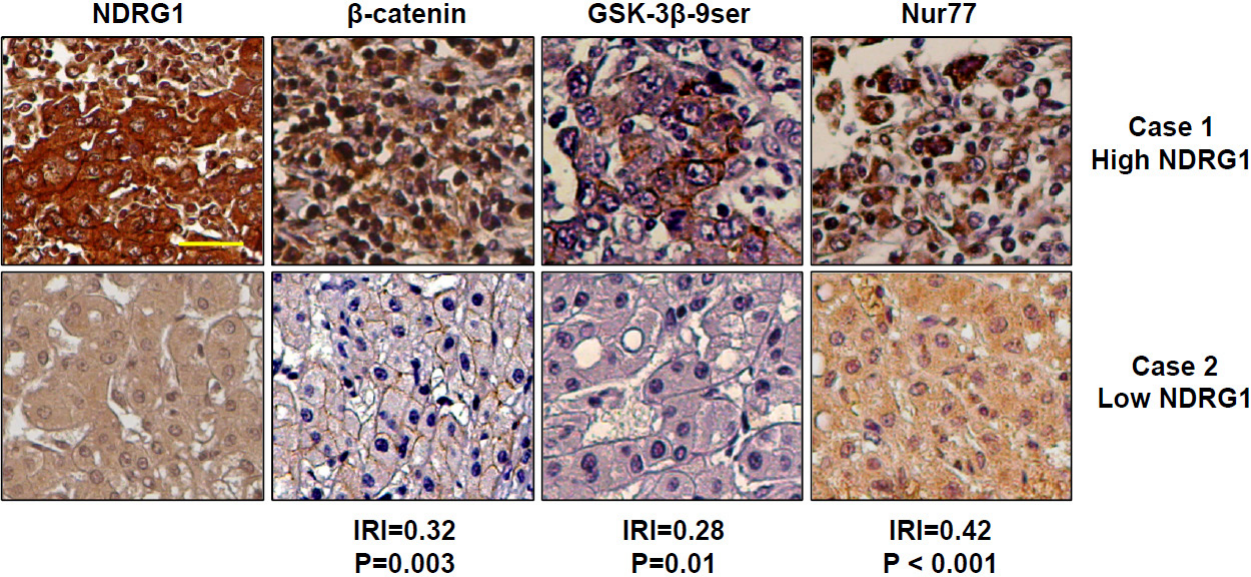
Expression of NDRG1, β-catenin, GSK-3β 9ser, and Nur77 in HCC patients Immunohistochemistry staining of NDRG1, β-catenin, GSK-3β 9ser and Nur77 were performed in 82 cases of HCC represented on tissue microarrays. Representative images of two HCC patients (Case 1 with high NDRG1, and Case 2 with low NDRG1) are shown for each protein. Based on semi-quantitative signal intensities (0-negative; 1- low expression, positive cells present in < 50% of entire area; 2- high expression, positive cells present in > 50% of entire area); correlation plots of NDRG1 with each protein shows that NDRG1 protein expression is positively correlated with β-catenin (│R│= 0.32, *p* = 0.003), GSK-3β 9ser (│R│= 0.28, *p* = 0.01), and Nur77 (│R│= 0.42, *p* < 0.001) (200× magnification; scale bars: 10 μm).

To determine the clinical significance of our *in vitro* and *in vivo* data, we examined the protein expression levels of NDRG1, GSK-3β, Nur77, and β-catenin in 82 cases of HCC patient specimens represented on tissue microarrays. Representative images are shown in Figure 7, and the detailed expression intensities of each protein in each patient specimen are listed in Supplementary Table 1). Semi-quantitative analysis based on immunohistochemical signal intensities indicated that NDRG1 expression was positively correlated with GSK-3β 9ser (│R│= 0.28, *p* = 0.01), Nur77 (│R│= 0.42, *p* < 0.001), and β-catenin (│R│= 0.32, *p* = 0.003) (Figure 7). The intensity of each protein expression has been shown in Supplementary Figure 2. Overall, HCC tissues with higher NDRG1 levels also have higher GSK-3β 9ser, Nur77, and β-catenin levels. This observation is consistent with our hypothesis that high levels of NDRG1 promote β-catenin accumulation *via* competitive binding of NDRG1 with GSK-3β and Nur77, thereby preventing its degradation.

## DISCUSSION

**Figure 8:**
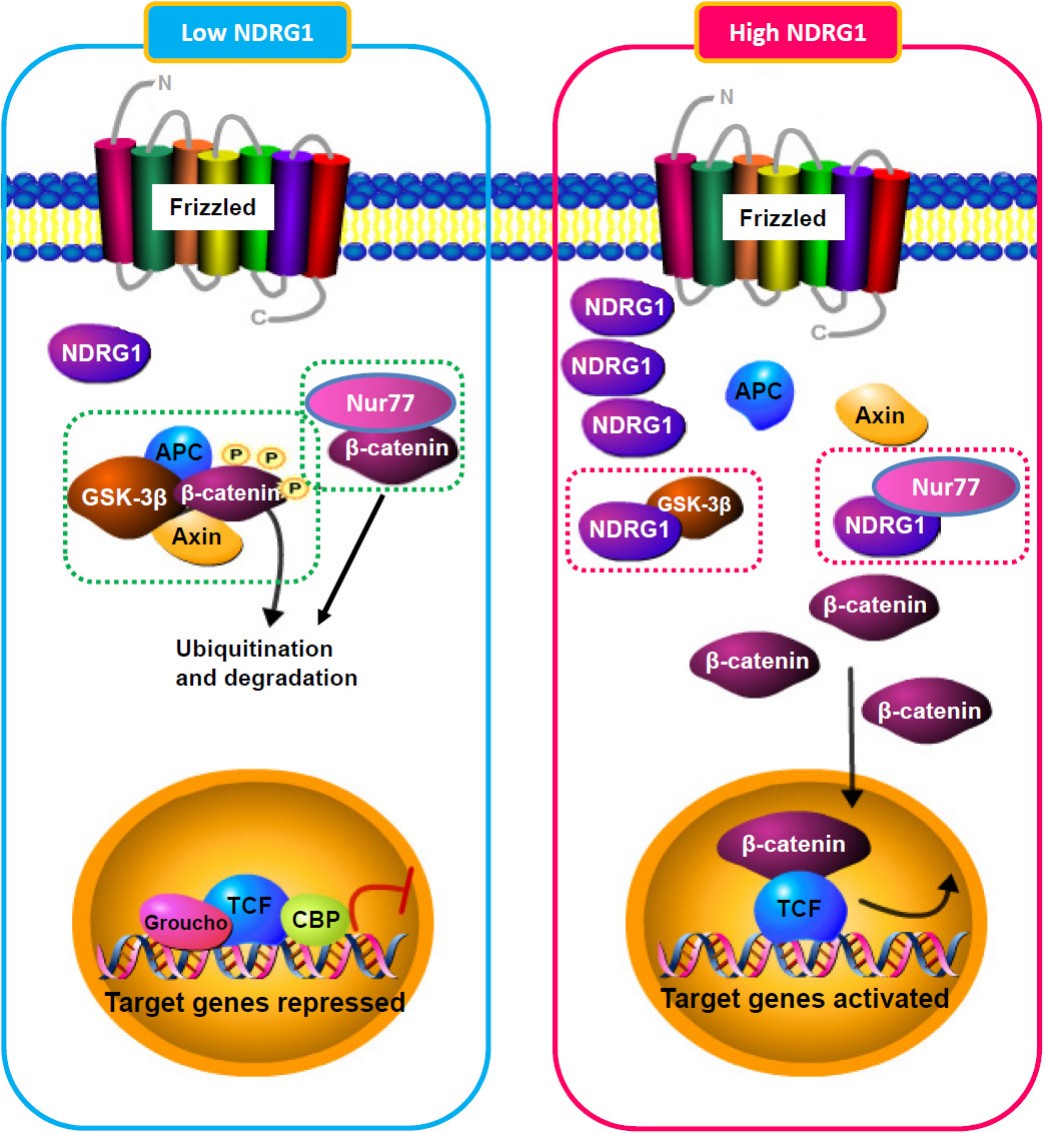
Schematic model illustrating the regulation of β-catenin turnover by NDRG1 and its interactions with GSK-3β and Nur77 in different cellular contexts (low or high NDRG1 levels)

The over-expression of NDRG1, a hypoxia-inducible protein, is associated with aggressive HCC phenotypes and with poor patient survival [5, 6]. To further understand the biological roles of NDRG1 in hepatocarcinogenesis, we used a protein microarray screen and identified GSK-3β and Nur77 as two novel interaction partners of NDRG1. Both GSK-3β and Nur77 mediate β-catenin degradation, suggesting a role of NDRG1 in β-catenin regulation. We showed that NDRG1 directly binds to GSK-3β and Nur77, which competitively disrupts binding of GSK-3β and Nur77 to β-catenin, thereby preventing β-catenin degradation and enhancing β-catenin accumulation in the nucleus (with subsequent activation of downstream events including up-regulation of oncogenic target genes such as Cyclin D1). Our results confirmed a mechanistic link between NDRG1 and the Wnt/β-catenin signaling pathway, and provide evidence that high levels of NDRG1 (either endogenously or induced by hypoxia) is associated with high levels of β-catenin accumulation and activity (Figure 8). This may in part account for over-activated Wnt/β-catenin signaling observed in HCC, which in turn promotes tumor progression and leads to aggressive tumor phenotypes.

Indeed, dysregulation of Wnt/β-catenin signaling pathway with nuclear accumulation of β-catenin has been associated with carcinogenesis [11]. In HCC, a whole-genome sequencing study of 88 pairs of matched HCC tumor/normal liver indicated that β-catenin is the most frequently mutated oncogene (15.9%) [12]. The cellular levels of β-catenin are tightly regulated by several distinct pathways including GSK-3β/APC/Axin1 [13], Nur77 [9] and p53/Siah-1/APC [14]. Many components of these destruction complexes are themselves frequently mutated in human cancer; therefore, novel approaches to reactivate these β-catenin destruction pathways may potentially have therapeutic significance in the multiple cancer types that present with over-activation of β-catenin. As an example, we demonstrated here that NDRG1 binds to both GSK-3β and Nur77 in HCC cells, resulting in reduced β-catenin degradation and subsequent nuclear accumulation; therefore approaches to inhibit these protein-protein interactions could potentially enhance β-catenin degradation and suppress β-catenin-mediated oncogenic activities with desirable anti-tumor effects.

NDRG1 has been reported to be involved in the Wnt/β-catenin signaling pathway. For example, NDRG1 was demonstrated to be a physiological substrate of GSK-3β [15], to interact directly with β-catenin [16], and with the Wnt co-receptor, LRP6 [17]. The functional consequences of such interactions appear to depend on specific cellular stresses, genetic and epigenetic backgrounds, and the tumor microenvironment. In prostate cancer, NDRG1 functions as a metastasis suppressor through its regulation of the Wnt/β-catenin pathway: (1) NDRG1 interacts with LRP6 to block Wnt/ β-catenin signaling which orchestrates a signaling network to impair metastasis [17]; (2) It activates GSK-3β by promoting its phosphorylation at Tyr279/216 to reduce total β-catenin levels [17]; (3) It suppresses the TGF-β induced epithelial-mesenchymal transition (EMT) through facilitating adherents complex formation by maintaining the cell membrane location of E-cadherin and β-catenin [18]; (4) It inhibits Wnt/β-catenin signaling through FRAT1 and PAK4 [19]. On the contrary, our observations demonstrate that NDRG1 is an oncogenic activator in HCC, possibly through its competitive binding with GSK-3β and Nur77 which reduces β-catenin degradation and enhances β-catenin transcriptional activation of its downstream oncogenes. In HCC specifically, the suppression of NDRG1 expression and function, including disruption of its interactions with GSK-3β and Nur77, are all potential means of therapeutic interventions, although in prostate cancer, the reverse may be beneficial. Thus, each cancer type needs to be evaluated individually for the appropriate intervention based on the specific roles of NDRG1 in that cancer.

Additionally, we show that regardless of the mutation status of *CTNNB1*, suppression of NDRG1 remains effective at reducing β-catenin levels and subsequent transcriptional activity. The cell lines used in this study represent different molecular subclasses of HCC, including HepG2 (which carries the 92 kDa wild-type β-catenin as well as the 73 kDa truncated β-catenin [20]), and Huh7 (which carries wild-type β-catenin) [21]. The truncated form of β-catenin has amino acids 25–140 deleted at N-terminal, which removes the GSK-3β binding site [20], causing accumulation of β-catenin in the cells. Thus, in HepG2 cells, truncated β-catenin does not interact with GSK-3β, but the wild-type β-catenin remains regulated *via* GSK-3β. Desbois-Mouthon *et al* reported that inhibition of GSK-3β through LiCl increased the amount of wild-type β-catenin in cytosolic extracts without modifying the level of truncated β-catenin [22]. Consistently, our study also demonstrated that suppression of NDRG1 caused GSK-3β activation only through a decrease in wild-type β-catenin without any effects on truncated β-catenin. Our data suggest that NDRG1 simultaneously interacts with two important controllers (GSK-3β and Nur77) of β-catenin, indicating its key involvement in regulating β-catenin levels and activity, and that the targeting of NDRG1 provides a broader and potentially more effective means of attenuating β-catenin-mediated oncogenic activities.

Like NDRG1, Nur77 (also called TR3 or NR4A1) is a hypoxia-inducible protein that plays either pro-malignant or anti-malignant roles depending on the cancer cell type and cellular environment [23]. Nur77 is an orphan member of the nuclear receptor superfamily that regulates many cellular processes, including proliferation, differentiation, and apoptosis of cancer cells [24]. Sun *et al* reported that Nur77 induced GSK-3β-independent degradation of β-catenin through direct interaction with β-catenin in the cytoplasm [9]. Furthermore, the Nur77 agonists, ATE-i2-b4 [9], H-9 [9], Cns-B [25] and its analogue Cns-A [26], suppressed both *in vitro* and *in vivo* growth of cancer cells through inducing cytoplasmic expression of Nur77 and its nuclear export [9]. In colon cancer, hypoxia was reported to enhance Nur77 and β-catenin expressions synergistically, stimulating colorectal cancer cell migration, invasion, and EMT [23]. We report that both Nur77 and NDRG1 are over-expressed in HCC cells and patient specimens, and that these two proteins interact to regulate β-catenin turnover. This interaction may potentially impact other cellular functions or signaling pathways regulated by either of these proteins, with resulting anti-tumor effects as observed by our *in vivo* data. Thus, further mechanistic studies of these two proteins in the context of HCC may be warranted, as they may offer new insights into hepatocarcinogenesis as well as therapeutic interventions.

The expression of NDRG1 in HCC is induced by hypoxia, through the HIF-1α transcription factor. We previously demonstrated that NDRG1 expression is associated with vascular invasion in HCC patients, and that it regulates cell invasion, which are molecular events mediated by hypoxia [6, 27]. We also showed that suppression of NDRG1 in HCC may indeed provide therapeutic benefits through its effects on cell viability, cell cycle progression, and cellular senescence [8]. By inference, the inhibition of HIF-1α is expected to inhibit NDRG1 expression and therefore also achieve these therapeutic effects. Sorafenib, a multiple tyrosine kinase inhibitor used as standard of care for advanced HCC patients, is known to inhibit HIF-1α synthesis [28]. We observed that sorafenib inhibited HIF-1α and NDRG1 expressions in Hep3B and HepG2 cells under hypoxia, concomitant with decreases in β-catenin, GSK-3β 9ser, Nur77 (nuclear only), and cyclin D1 levels (Supplementary Figure 3). The cytoplasmic levels of Nur77 were enhanced. Thus, the anti-tumor efficacy of sorafenib may in part be mediated through its indirect inhibition of NDRG1 expression and functions. Indeed, sorafenib was reported to decrease Wnt/β-catenin activity in HCC cells *in vitro*, and to partially disrupt the activation of Wnt/β-catenin pathway in HCC xenografts [29]. It was also demonstrated to sensitize HCC cells to cisplatin treatment through inhibition of Wnt/β-catenin signaling [30]. Sorafenib therefore represents a pharmacological means to indirectly inhibit the expression and functions of NDRG1, and suggests that sorafenib may be combined with other NDRG1-based therapeutic interventions to achieve synergistic anti-tumor effects in advanced HCC patients with aggressive phenotypes.

In conclusion, we present new data that NDRG1 regulates the Wnt/β-catenin signaling pathway in HCC cells, through its direct interactions with GSK-3β and Nur77 to prevent β-catenin degradation. The disruption of protein-protein interactions, which are signaling hubs that link and transmit oncogenic signals across various molecular networks, offer a potentially effective means of halting oncogenic signaling (e. g. inhibition of p53 and MDM2 interaction by Nutlins) [31, 32]. Our data allows us to further understand the biological processes underlying NDRG1-associated tumor aggressiveness, and also suggests new avenues for developing therapeutic approaches for HCC, such as through the disruption of NDRG1 interaction with GSK-3β and/or Nur77.

## MATERIALS AND METHODS

### Cell lines and cell culture

The HCC cell lines HepG2, Hep3B, Huh7, PLC/PRF/5, HepG2-*Luc*(+) and Hep3B-*Luc*(+) were maintained in high-glucose DMEM supplemented with 10% fetal bovine serum (Invitrogen, Carlsbad, CA), and 1% penicillin-streptomycin. The cells were incubated in a humidified atmosphere containing 5% CO_2_ at 37°C. Where appropriate, cells were treated with the chemicals LY294002 (LY) (Cell Signaling Technology Inc., Danvers, MA) [33]; or Cns-A (Cytosporone A n-amyl ester, compound 10i) (Sigma-Aldrich, St. Louis, MO) [26] at 10 μM final concentration, for 48 hours.

### Cloning, protein purification, and protein microarray screening

Full length NDRG1 protein was amplified from pcDNA 3.0 NDRG1 vector using the following primers: upstream (5′- TTT GGA TCC GGT AAG CCT ATC CCT AAC CCT CTC CTC GGT CTC GAT TCT ACG ATG TCT CGG GAG ATG CAG GAT G -3′), and downstream (5′- TTT CTC GAG CTA GCA GGA GAC CTC CAT GGA CTT G -3′). The PCR reaction was performed in a GeneAmp PCR system 2700 (Applied Biosystems, Foster City, CA) under the following conditions: heat activation of the polymerase for 5 min at 95°C, followed by 30 cycles of 95°C for 30 sec, 58°C for 30 sec, and 72°C for 2 min; with a final extension at 72°C for 10 min. The PCR-amplified product of the NDRG1 coding region is constructed into the BamHI and XhoI sites of vector pet28A (Merck Biosciences, Darmstadt, Germany). Recombinant V5-NDRG1 peptide was generated in E. coli (strain BL21, DE3) with IPTG induction, and native NDRG1 protein was purified using Ni-NTA Superflow Agarose (Invitrogen, Carlsbad, CA) following the manufacturer's protocol. The concentration of V5-NDRG1 protein was measured by the BCA Protein Assay (Pierce, Rockford, IL). Purified V5-NDRG1 protein was confirmed using Coomassie blue staining and Western blotting using V5-HRP secondary antibody (Invitrogen, Carlsbad, CA).

The purified V5-NDRG1 protein was used to probe the Human ProtoArray^®^ protein microarray v5.0 (Invitrogen, Carlsbad, CA) according to the manufacturer's protocol. Briefly, ProtoArray slides were pre-blocked with blocking buffer, and then incubated for 90 min at 4°C with 1 μg V5-NDRG1 diluted in PBS buffer, with gentle shaking. Slides were then washed four times with 5 ml PBS. Alexa Fluor^®^ 647 Anti-V5 Antibody (1 μg/mL; Invitrogen, Carlsbad, CA) was then added and incubated for 90 min at 4°C with gentle shaking. Slides were then washed again four times with PBS, and dried. Dried slides were scanned using GenePix 4000B (Molecular Devices Corporation, Sunnyvale, CA), and images analyzed using ProtoArray Prospector (Invitrogen, Carlsbad, CA).

### Protein extraction and Immunoblotting

For Western blotting, cells were lysed with T-PER Tissue Protein Extraction Reagent (Thermo Fisher Scientific Inc., Waltham, MA). Protein lysates suspended in loading buffer were resolved *via* SDS-polyacrylamide gel electrophoresis and blotted onto a nitrocellulose membrane. The membrane was incubated with the desired primary antibody, followed by incubation with appropriate HRP-conjugated secondary antibodies. The protein expression was detected by SuperSignal West Pico Chemiluminescent Substrate (Thermo Fisher Scientific Inc., Waltham, MA) according to the manufacturer's protocol. Primary antibodies used are: NDRG1, Nur77 (Abcam, Cambridge, UK); p-Rb, GSK-3β, GSK-3β-9ser, Histone 3 (Cell Signaling Technology Inc., Danvers, MA); β-catenin, p16, GAPDH (Santa Cruz Biotechnology, Santa Cruz, CA). Secondary antibodies used are: goat anti-rabbit and goat anti-mouse (Santa Cruz Biotechnology, Santa Cruz, CA).

### Co-immunoprecipitation assay

Total protein (500 μg) in cell lysates were incubated with 2 μg anti-NDRG1 or anti-β-catenin antibody at 4°C for 2 hours; 20 μl A/G PLUS-Agarose beads (Santa Cruz Biotechnology, Santa Cruz, CA) were then added into each sample and incubated at 4°C overnight. After incubation, the beads were separated from the lysis buffer and washed three times in cold PBS. Immunocomplexes were resuspended in 2x loading buffer, heat-denatured, and centrifuged; supernatants were collected and resolved by SDS-PAGE on a 10% polyacrylamide gel, and immunoblotting was performed with antibodies as indicated.

### Immunofluorescence staining

Cells were seeded on the chamber slide and fixed with ice cold 4% paraformaldehyde in PBS for 20 min, permeabilized with 0.1% Triton X-100 for 15 min, and blocked with 1% BSA for one hour. Cells were stained for indicated primary antibodies and secondary antibody, and then visualized using an Eclipse E600 image analysis system (Nikon, Tokyo, Japan).

### Induction of hypoxia

Cells were plated at the desired density 24 hours prior to placement in a hypoxia chamber maintained at 0.5% oxygen for desired time periods depending on the experiment. Alternatively, hypoxia was induced chemically by incubating the plated cells in GasPak pouches for anaerobic culture (BD Biosciences, San Jose, CA) according to the manufacturer's instructions.

### siRNA transfection

Target specific siRNA (20 nM) against NDRG1 (Ambion, Austin, TX) was transfected into cells in 6-well plates using Lipofectamine 2000 (Invitrogen, Carlsbad, CA) according to the manufacturer's instructions. The siRNA sequences are: NDRG1 siRNA: 5- GCU GAU CCA GUU UCC GGA Att-3.

### Immunohistochemistry of patient samples and mouse xenografts

Clinical samples were collected from HCC patients who underwent hepatectomy at Stanford Hospital (Palo Alto, California, USA). This study was approved by the Institutional Review Board at Stanford University, and all patients signed informed consent forms prior to tissue collection. Immunohistochemical staining was performed following standard protocol as previously described [8]. Sections were developed using Dako EnVision System and were then counterstained with hematoxylin (Dako, Glostrup, Denmark). Images were viewed with Nikon epifluorescent upright microscope E600 (Nikon, Tokyo, Japan). Slides were scored based on the signal intensities (0 – negative; 1– low expression, positive cells present in < 50% of the entire area; 2– high expression, positive cells present in > 50% of the entire area). Xenograft tumor tissues were collected from mice and stained following standard immunohistochemical protocol [8].

### Subcutaneous xenografts in nude mice

HepG2-*Luc*(+) and Hep3B-*Luc*(+) cells (1 × 10^6^ each) stably transfected with control-shRNA or NDRG1-shRNA were injected into 4 weeks old nude mice (Charles River Laboratories International Inc., Wilmington, MA) to induce subcutaneous tumor formation. Cells with control-shRNA were injected into the left shoulders and cells with NDRG1-shRNA were injected into the right shoulders (*n* = 8 for each group). Doxycycline (200 μg/ml) (Sigma-Aldrich, St. Louis, MO) was added daily into the drinking water of nude mice. Whole body luminescence intensity and tumor size were measured weekly. After 4 weeks, the mice were sacrificed and tumor tissues harvested for further investigations.

### Data analysis

Statistical analyses were performed using PRISM version 5.0 software (GraphPad Software, Inc., La Jolla, CA). The Student's *t*-test and One-way ANOVA were used to calculate statistical significance. The correlation coefficient was calculated to measure the linear association among two sets of variables. Statistical significance is indicated by *p* < 0.05.

## SUPPLEMENTARY MATERIAL FIGURES AND TABLE



## Abbreviations

NDRG1: N-Myc downstream regulated gene 1
HCC: Hepatocellular carcinoma
GSK-3β: glycogen synthase kinase-3β
Nur77: Orphan nuclear receptor

## References

[1] Sibold S, Roh V, Keogh A, Studer P, Tiffon C, Angst E, Vorburger SA, Weimann R, Candinas D, Stroka D (2007). Hypoxia increases cytoplasmic expression of NDRG1, but is insufficient for its membrane localization in human hepatocellular carcinoma. Febs Letters.

[2] Cangul H (2004). Hypoxia upregulates the expression of the NDRG1 gene leading to its overexpression in various human cancers. BMC Genet.

[3] Wang Q, Li LH, Gao GD, Wang G, Qu L, Li JG, Wang CM (2013). HIF-1alpha up-regulates NDRG1 expression through binding to NDRG1 promoter, leading to proliferation of lung cancer A549 cells. Molecular Biology Reports.

[4] Jung EU, Yoon JH, Lee YJ, Lee JH, Kim BH, Yu SJ, Myung SJ, Kim YJ, Lee HS (2010). Hypoxia and retinoic acid-inducible NDRG1 expression is responsible for doxorubicin and retinoic acid resistance in hepatocellular carcinoma cells. Cancer Letters.

[5] Chen X, Cheung ST, So S, Fan ST, Barry C, Higgins J, Lai KM, Ji J, Dudoit S, Ng IO, Van De Rijn M, Botstein D, Brown PO (2002). Gene expression patterns in human liver cancers. Mol Biol Cell.

[6] Chua MS, Sun H, Cheung ST, Mason V, Higgins J, Ross DT, Fan ST, So S (2007). Overexpression of NDRG1 is an indicator of poor prognosis in hepatocellular carcinoma. Mod Pathol.

[7] Cheng J, Xie HY, Xu X, Wu J, Wei XY, Su R, Zhang W, Lv Z, Zheng SS, Zhou L (2011). NDRG1 as a biomarker for metastasis, recurrence and of poor prognosis in hepatocellular carcinoma. Cancer Letters.

[8] Lu WJ, Chua MS, So SK (2014). Suppressing N-Myc downstream regulated gene 1 reactivates senescence signaling and inhibits tumor growth in hepatocellular carcinoma. Carcinogenesis.

[9] Sun Z, Cao X, Jiang MM, Qiu Y, Zhou H, Chen L, Qin B, Wu H, Jiang F, Chen J, Liu J, Dai Y, Chen HF, Hu QY, Wu Z, Zeng JZ (2012). Inhibition of beta-catenin signaling by nongenomic action of orphan nuclear receptor Nur77. Oncogene.

[10] Nakamura T, Hamada F, Ishidate T, Anai K, Kawahara K, Toyoshima K, Akiyama T (1998). Axin, an inhibitor of the Wnt signalling pathway, interacts with beta-catenin, GSK-beta and APC and reduces the beta-catenin level. Genes to Cells.

[11] Pez F, Lopez A, Kim M, Wands JR, Caron de Fromentel C, Merle P (2013). Wnt signaling and hepatocarcinogenesis: molecular targets for the development of innovative anticancer drugs. Journal of Hepatology.

[12] Kan Z, Zheng H, Liu X, Li S, Barber TD, Gong Z, Gao H, Hao K, Willard MD, Xu J, Hauptschein R, Rejto PA, Fernandez J, Wang G, Zhang Q, Wang B (2013). Whole-genome sequencing identifies recurrent mutations in hepatocellular carcinoma. Genome Research.

[13] Ikeda S, Kishida S, Yamamoto H, Murai H, Koyama S, Kikuchi A (1998). Axin, a negative regulator of the Wnt signaling pathway, forms a complex with GSK-3beta and beta-catenin and promotes GSK-3beta-dependent phosphorylation of beta-catenin. The EMBO Journal.

[14] Liu J, Stevens J, Rote CA, Yost HJ, Hu Y, Neufeld KL, White RL, Matsunami N (2001). Siah-1 mediates a novel beta-catenin degradation pathway linking p53 to the adenomatous polyposis coli protein. Molecular Cell.

[15] Murray JT, Campbell DG, Morrice N, Auld GC, Shpiro N, Marquez R, Peggie M, Bain J, Bloomberg GB, Grahammer F, Lang F, Wulff P, Kuhl D, Cohen P (2004). Exploitation of KESTREL to identify NDRG family members as physiological substrates for SGK1 and GSK3. Biochemical Journal.

[16] Tu LC, Yan X, Hood L, Lin B (2007). Proteomics analysis of the interactome of N-myc downstream regulated gene 1 and its interactions with the androgen response program in prostate cancer cells. Mol Cell Proteomics.

[17] Liu W, Xing F, Iiizumi-Gairani M, Okuda H, Watabe M, Pai SK, Pandey PR, Hirota S, Kobayashi A, Mo YY, Fukuda K, Li Y, Watabe K (2012). N-myc downstream regulated gene 1 modulates Wnt-beta-catenin signalling and pleiotropically suppresses metastasis. EMBO Mol Med.

[18] Chen Z, Zhang D, Yue F, Zheng M, Kovacevic Z, Richardson DR (2012). The iron chelators Dp44mT and DFO inhibit TGF-beta-induced epithelial-mesenchymal transition via up-regulation of N-Myc downstream-regulated gene 1 (NDRG1). J Biol Chem.

[19] Jin R, Liu W, Menezes S, Yue F, Zheng M, Kovacevic Z, Richardson DR (2014). The metastasis suppressor NDRG1 modulates the phosphorylation and nuclear translocation of beta-catenin through mechanisms involving FRAT1 and PAK4. Journal of Cell Science.

[20] de La Coste A, Romagnolo B, Billuart P, Renard CA, Buendia MA, Soubrane O, Fabre M, Chelly J, Beldjord C, Kahn A, Perret C (1998). Somatic mutations of the beta-catenin gene are frequent in mouse and human hepatocellular carcinomas. Proceedings of the National Academy of Sciences of the United States of America.

[21] Lachenmayer A, Alsinet C, Savic R, Cabellos L, Toffanin S, Hoshida Y, Villanueva A, Minguez B, Newell P, Tsai HW, Barretina J, Thung S, Ward SC, Bruix J, Mazzaferro V, Schwartz M (2012). Wnt-pathway activation in two molecular classes of hepatocellular carcinoma and experimental modulation by sorafenib. Clinical Cancer Research.

[22] Desbois-Mouthon C, Cadoret A, Blivet-Van Eggelpoel MJ, Bertrand F, Cherqui G, Perret C, Capeau J (2001). Insulin and IGF-1 stimulate the beta-catenin pathway through two signalling cascades involving GSK-3beta inhibition and Ras activation. Oncogene.

[23] To SK, Zeng WJ, Zeng JZ, Wong AS (2014). Hypoxia triggers a Nur77-beta-catenin feed-forward loop to promote the invasive growth of colon cancer cells. British Journal of Cancer.

[24] Zhang XK (2007). Targeting Nur77 translocation. Expert Opinion on Therapeutic Targets.

[25] Zhan YP, Du XP, Chen HZ, Liu JJ, Zhao BX, Huang DH, Li GD, Xu QY, Zhang MQ, Weimer BC, Chen D, Cheng Z, Zhang LR, Li QX, Li SW, Zheng ZH (2008). Cytosporone B is an agonist for nuclear orphan receptor Nur77. Nat Chem Biol.

[26] Liu JJ, Zeng HN, Zhang LR, Zhan YY, Chen Y, Wang Y, Wang J, Xiang SH, Liu WJ, Wang WJ, Chen HZ, Shen YM, Su WJ, Huang PQ, Zhang HK, Wu Q (2010). A unique pharmacophore for activation of the nuclear orphan receptor Nur77 *in vivo* and *in vitro*. Cancer Research.

[27] Yan X, Chua MS, Sun H, So S (2008). N-Myc down-regulated gene 1 mediates proliferation, invasion, and apoptosis of hepatocellular carcinoma cells. Cancer Letters.

[28] Liu LP, Ho RL, Chen GG, Lai PB (2012). Sorafenib inhibits hypoxia-inducible factor-1alpha synthesis: implications for antiangiogenic activity in hepatocellular carcinoma. Clinical Cancer Research.

[29] Lachenmayer A, Alsinet C, Savic R, Cabellos L, Toffanin S, Hoshida Y, Villanueva A, Minguez B, Newell P, Tsai HW, Barretina J, Thung S, Ward SC, Bruix J, Mazzaferro V, Schwartz M (2012). Wnt-pathway activation in two molecular classes of hepatocellular carcinoma and experimental modulation by sorafenib. Clinical Cancer Research.

[30] Wei Y, Shen N, Wang Z, Yang G, Yi B, Yang N, Qiu Y, Lu J (2013). Sorafenib sensitizes hepatocellular carcinoma cell to cisplatin via suppression of Wnt/beta-catenin signaling. Mol Cell Biochem.

[31] Vassilev LT, Vu BT, Graves B, Carvajal D, Podlaski F, Filipovic Z, Kong N, Kammlott U, Lukacs C, Klein C, Fotouhi N, Liu EA (2004). *In vivo* activation of the p53 pathway by small-molecule antagonists of MDM2. Science.

[32] Lu WJ, Lee NP, Kaul SC, Lan F, Poon RT, Wadhwa R, Luk JM (2011). Mortalin-p53 interaction in cancer cells is stress dependent and constitutes a selective target for cancer therapy. Cell Death and Differentiation.

[33] Beurel E, Kornprobst M, Blivet-Van Eggelpoel MJ, Cadoret A, Capeau J, Desbois-Mouthon C (2005). GSK-3beta reactivation with LY294002 sensitizes hepatoma cells to chemotherapy-induced apoptosis. International Journal of Oncology.

