# Supplementary material for: NDRG1 promotes growth of hepatocellular carcinoma cells by directly interacting with GSK-3β and Nur77 to prevent β-catenin degradation
Source: Oncotarget. 2015 Aug 20;6(30):29847–59. doi: 10.18632/oncotarget.4913 (PMC4745767; doi:10.18632/oncotarget.4913)
Supplement: Supplementary file 1 [file oncotarget-06-29847-s001.pdf]

SUPPLEMENTARY FIGURES AND TABLE

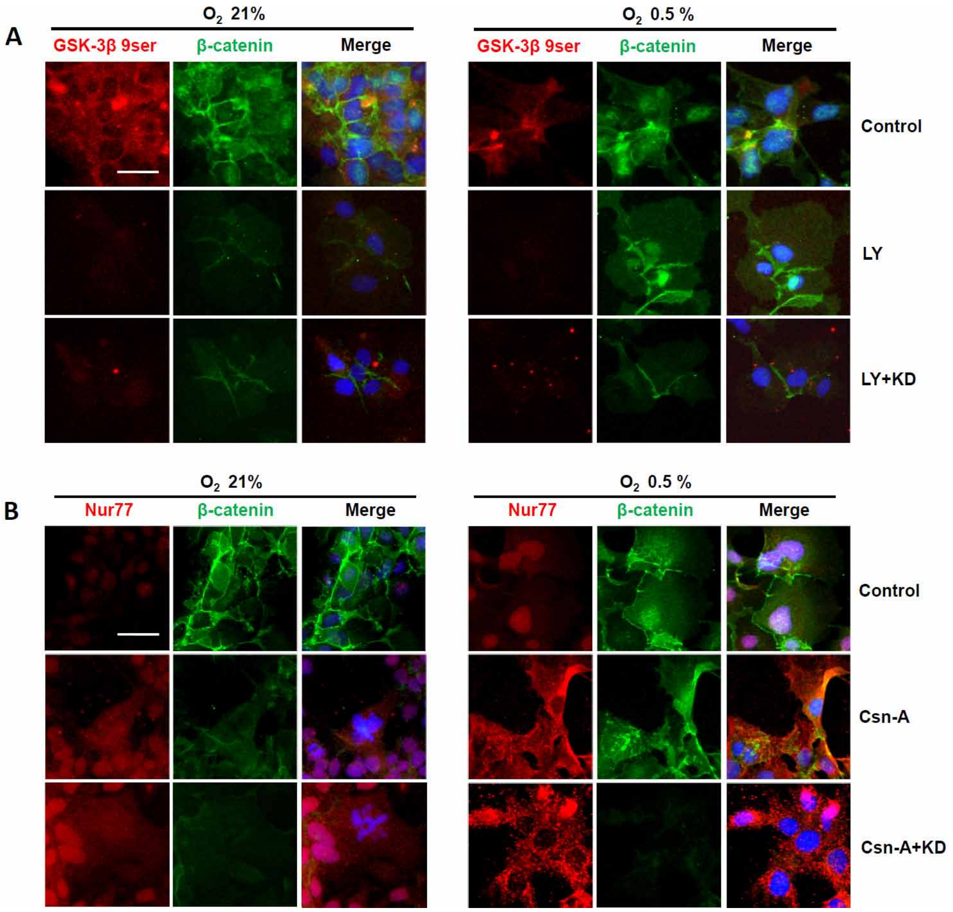

Supplementary Figure S1: Suppression of NDRG1 facilitates degradation of β-catenin triggered by GSK-3β agonist or Nur77 agonist or in Hep3B cells under normoxia and hypoxia. Immunofluorescence staining for protein expression of β-catenin, NDRG1, GSK-3β 9ser, and Nur77, showing that suppression of NDRG1 enhanced β-catenin degradation in Hep3B cells treated with either **A.** GSK-3β agonist or **B.** Nur77 agonist under normoxia or hypoxia when compared to untreated control group (400× magnification; scale bars: 5 μm).

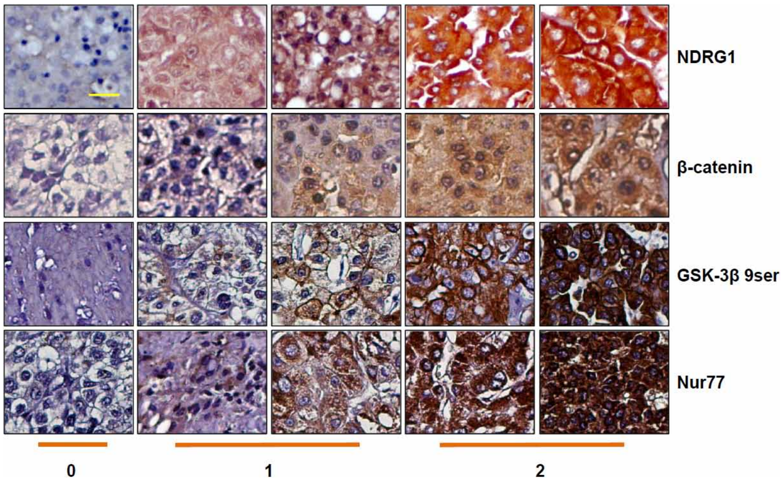

Supplementary Figure S2: Representative immunohistochemistry images showing the signal intensities of NDRG1, β-catenin, GSK-3β, and Nur77 in HCC tissues. Based on the signal intensities of immunohistochemical staining, sections were divided into three groups (0 – negative; 1 – low expression, positive cells present in < 50% of the entire area; 2 – high expression, positive cells present in > 50% of the entire area) (400× magnification; scale bars: 5 µm).

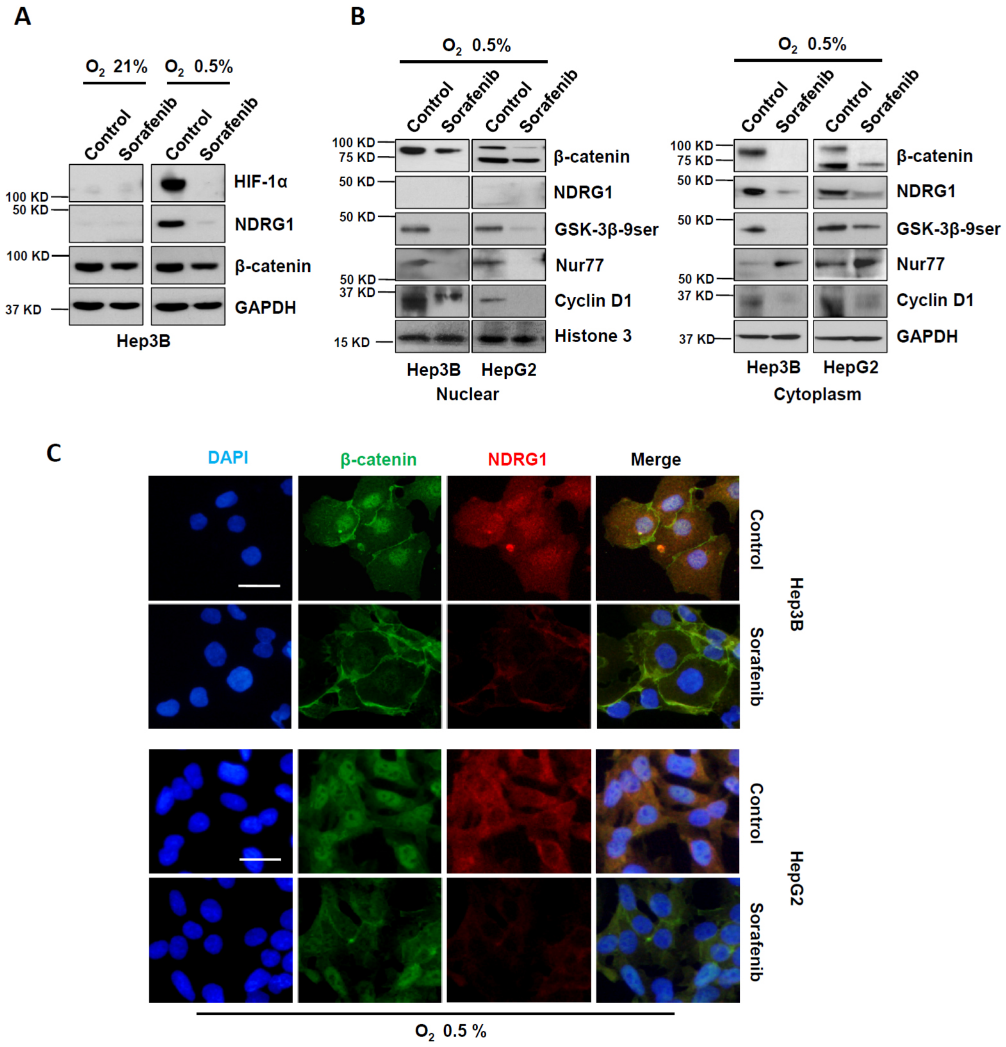

Supplementary Figure S3: Inhibition of NDRG1 by sorafenib prevented β-catenin nuclear accumulation in HCC cells under hypoxia. A. Western blot detected HIF-1α, NDRG1, and total β-catenin expression in Hep3B cells under normoxia and hypoxia after treatment with 5 μM sorafenib for 48 hours. **B.** Sorafenib decreased nuclear β-catenin accumulation in HCC cells when treated under hypoxia. Corresponding changes in GSK-3β 9ser, Nur77, and Cyclin D1 are also shown. GAPDH and Histone3 are used as loading controls. **C.** Immunofluorescence staining detected NDRG1 and β-catenin localizations in HCC cells treated with sorafenib under hypoxia (400× magnification; scale bars: 5 μm upper panel, 3 μm lower panel).

Supplementary Table S1: Immunohistochemistry scores[a] for NDRG1, β-catenin, GSK-3β-9ser, and Nur77 expression levels in 82 HCC patients

Patient No.	Expression intensity			
	NDRG1	β-catenin	GSK-3β-9ser	Nur77
1	0	0	0	0
2	1	1	0	1
3	1	1	0	2
4	1	0	2	1
5	1	1	1	2
6	1	1	0	1
7	1	0	0	2
8	1	0	1	1
9	1	1	2	1
10	1	1	1	1
11	1	0	1	2
12	1	2	0	2
13	1	0	1	1
14	1	1	0	1
15	1	1	2	2
16	1	1	1	1
17	1	1	1	1
18	1	1	1	1
19	1	1	1	1
20	1	1	2	2
21	1	1	1	2
22	1	1	2	1
23	1	0	1	1
24	2	1	1	2
25	2	0	1	0
26	2	1	1	2
27	2	1	1	2
28	2	2	1	1
29	2	1	1	2
30	2	1	1	2
31	2	1	2	1
32	2	1	0	2
33	2	1	1	2
34	2	1	2	1

(*Continued*)

Patient No.	Expression intensity			
	NDRG1	β-catenin	GSK-3β-9ser	Nur77
35	2	1	2	1
36	2	2	1	1
37	2	1	1	2
38	2	1	0	2
39	2	1	2	2
40	2	1	1	2
41	2	2	1	2
42	2	2	2	2
43	2	1	1	1
44	2	2	2	2
45	2	1	1	2
46	2	0	1	2
47	2	1	1	2
48	2	2	2	2
49	2	0	2	2
50	2	0	1	2
51	2	1	2	1
52	2	1	1	2
53	2	1	0	1
54	2	2	2	2
55	2	2	1	1
56	2	2	0	2
57	2	0	1	2
58	2	1	1	1
59	2	1	0	2
60	2	1	1	2
61	2	2	1	2
62	2	1	1	2
63	2	0	2	2
64	2	2	0	1
65	2	2	2	2
66	2	1	2	2
67	2	1	1	2
68	2	2	2	2
69	2	2	1	2
70	2	1	1	2

(*Continued*)

Patient No.	Expression intensity			
	NDRG1	β-catenin	GSK-3β-9ser	Nur77
71	2	2	2	2
72	2	1	2	2
73	2	1	1	2
74	2	1	1	2
75	2	0	2	2
76	2	1	1	2
77	2	2	1	2
78	2	2	1	2
79	2	2	2	1
80	2	2	2	1
81	2	1	2	2
82	2	0	1	2

[a] 0 – negative; 1– low expression, positive cells present in < 50% of the entire area; 2– high expression, positive cells present in > 50% of the entire area.
